# Imagining Speeds up the Effect of Motor Imagery on Central Motor Conduction Time

**DOI:** 10.7759/cureus.71798

**Published:** 2024-10-18

**Authors:** H. Evren Boran, Merve Ceren Akgor, Ozlem Kurtkaya Kocak, Halil Can Alaydin, Hasan Kilinc, Nur Turkmen, Bulent Cengiz

**Affiliations:** 1 Department of Neurology, Gazi University Faculty of Medicine, Ankara, TUR; 2 Department of Clinical Neurophysiology, Gazi University Faculty of Medicine, Ankara, TUR; 3 Department of Brain Stimulation and Motor Control, Neuroscience and Neurotechnology Center of Excellence (NOROM), Ankara, TUR; 4 Department of Neurology, Ankara Bilkent City Hospital, Ankara, TUR

**Keywords:** central motor conduction time, motor evoked potential amplitude, motor evoked potential latency, motor imagery, transcranial magnetic stimulation (tms)

## Abstract

Introduction: Although motor imagery (MI) has been reported to increase motor cortical excitability, its effect on central motor conduction time (CMCT), a widely used neurophysiological diagnostic method, has not been investigated. In this study, we sought to determine the effect of MI on CMCT.

Methods: In this cross-sectional study, 21 healthy volunteers (11 females, 10 males) aged 24 to 67 years (mean age: 38.8 years) were recruited between April 2022 and June 2023. CMCT was calculated during MI from the abductor digiti minimi (ADM) and tibialis anterior (TA) muscles. Measurements were also performed with conventional measurement methods, such as resting and voluntary contraction, to compare the effect of MI on CMCT.

Results: The ANOVA test revealed that the CMCT session (rest, MI, and voluntary contraction) was a significant factor (p < 0.05). In both muscles, CMCT was shorter in the imagery state than in the resting state but longer than in the voluntary contraction state (p < 0.05). Similarly, motor-evoked potential (MEP) latencies obtained during imagery were shorter for both muscles than the resting state but longer for the voluntary contraction state.

Conclusion: The study’s findings suggest that MI is a mental activity that modulates CMCT measurement. MI shows a voluntary contraction-like effect on CMCT and MEP latency, although the effect is more uncertain.

## Introduction

Transcranial magnetic stimulation (TMS) is a non-invasive brain stimulation method based on the principle of stimulating the cortex by generating electromagnetic current through a coil over the scalp [1]. Motor cortex excitability and the function of inhibitory and facilitatory circuits can be evaluated with TMS [2]. Due to this feature, TMS is used for diagnostic purposes. The most used TMS parameter for diagnostic purposes is central motor conduction time (CMCT). CMCT provides information about the integrity of the corticospinal tract [3]. It is decided whether there is corticospinal and corticobulbar tract involvement based on CMCT normal values for the lower extremity, upper extremity, and bulbar muscles. CMCT is prolonged in many diseases, such as multiple sclerosis (MS), amyotrophic lateral sclerosis (ALS), stroke, and myelopathy [4].

CMCT is easy to apply but contains important points to consider. The most important is whether the muscle being recorded is contracting or not. Voluntary contraction increases spinal motor neuron excitability and shortens CMCT [5]. Therefore, when using normative values, it is important whether the obtained data are from the muscle at rest or under voluntary contraction.

Motor imagery (MI) is a mental activity in which a movement is performed mentally, but there is no motor output or muscle contraction. Studies have shown that the centres executing the imagined movement are activated during MI [6]. Due to this feature, MI is used as a motor system activation tool in athletes and musicians and for rehabilitation purposes for diseases [7].

The present study was planned to investigate a previously unexplored topic, the effect of MI, which causes activation in the motor system, on the CMCT. We hypothesize that during MI, as during voluntary contraction, the CMCT will be shortened.

## Materials and methods

The study was conducted at Gazi University Faculty of Medicine, Department of Neurology, Neurophysiology Division, Motor Control Laboratory. The local Ethics Committee of Gazi University approved the study. Volunteers were informed in detail about the purpose and scope of the study based on the Informed Voluntary Consent Form. Their consent and signature were obtained.

Participants

The a priori sample size was first calculated using G*Power statistical software (Ver. 3.1.9.6 Heinrich-Heine-Universität Düsseldorf, Düsseldorf, Germany) with the following input parameters: α = 0.05, 1 - β = 0.80, and f = 0.30 [8]. The analysis showed that the minimum requirement for this study was 20 participants. Between April 2022 and June 2023, 21 healthy volunteers (11 females, 10 males) aged between 24 and 67 years (mean age: 38.8 years) with a mean height of 169.7 cm (158-188 cm) without any neurological symptoms or examination findings and without contraindications for TMS were included in the study. Volunteers were asked not to consume alcohol for 24 hours and coffee for 12 hours before the study.

Peripheral nerve stimulation

This study examined the right side of all volunteers. Volunteers were seated on a comfortable chair. Central conduction time was calculated according to the F method. For F response, 20 stimuli were given and the minimum F latency was measured. A Nihon Kohden electromyography (EMG) device, Neuropack MEB- 5504 K, (Nihon Kohden Corporation, Tokyo, Japan), was used to study F and M responses. The Ag-AgCl surface electrodes were used for recording. The recording electrodes were placed with the active electrode on the muscle belly while the reference was on the tendon area. Electrical stimulation was applied with a surface-stimulating electrode. The ulnar nerve was stimulated at the wrist and the peroneal nerve at the knee at supramaximal stimulus intensity, which was a square-wave electrical pulse with a 0.1 ms duration. The EMG signal was band pass filtered at 3-10 KHz.

Central motor conduction time

TMS was performed as monophasic stimulation with Neurosoft stimulator (Neuro-MSX, Ivanovo, Russia) using Neurosoft FEC-02-100 figure-of-eight coil for abductor digiti minimi (ADM) muscle and RC-02-150 ring coil for tibialis anterior (TA) muscle. First, the region (hotspot) where the largest amplitude motor-evoked potential (MEP) responses were obtained stably for the muscle under study was determined. This region was marked on the cap worn by the volunteer and the coil was kept constant during this study based on these marks. Resting motor threshold (RMT) was calculated for each muscle as the lowest TMS stimulus intensity that produced MEP of ≥ 50 μV in at least 5 of 10 stimuli. TMS was given at 120% RMT intensity for the CMCT protocol. MEP responses obtained with 10 TMS at 4-5 s intervals for each muscle were recorded. The shortest latency among 10 MEP responses was accepted as the MEP latency for that muscle.

Central Motor Conduction Time at Rest

CMCT was performed when the muscle was completely at rest.

C*entral Motor Conduction Time in Mild Contraction*

The volunteer was asked to perform the muscle at approximately 20% of its maximal contraction. To maintain this contraction level, the volunteers were visually monitored using an EMG signal.

Central Motor Conduction Time During Imagery

In this protocol, the volunteer was asked to imagine performing a simple motor movement related to the relevant muscle but not to perform any movement. For ADM muscle, the volunteer was asked to visually imagine moving his/her index finger a few centimetres laterally. For TA muscle, the volunteer was asked to visually imagine lifting the toes a few centimetres with the heel on the ground.

To ensure volunteers were at rest, 200 ms before TMS was included in the total MEP recording time. CMCT protocol order was determined randomly for each volunteer.

CMCT was calculated with the formula:

$\text{CMCT} = \text{MEP Latency - } \left( \frac{\displaystyle \text{M Latency + F Latency - 1}}{\displaystyle \text{2}} \right)$ 

Signals were digitised using a CED micro1401 laboratory interface (Cambridge Electronic Design, Cambridge, England, UK) and analysed offline using Signal software (Cambridge Electronic Design, Cambridge, England, UK).

Statistical analysis

The distribution of the data was evaluated using the Shapiro-Wilk test. Data not normally distributed were logarithmically transformed. Mean MEP amplitude, MEP latency, and CMCT performed in three different conditions were calculated for each muscle. Correlation analysis was performed using Pearson's method. The effect of the sessions on the parameters was evaluated with a repeated ANOVA test (rest x voluntary contraction and MI). The statistical significance level was accepted as p < 0.05. The R Version 4.0.2 (The R Foundation for Statistical Computing, Vienna, Austria) programme was used for statistical analysis.

## Results

MEP amplitude values that did not show normal distribution were subjected to logarithmic transformation. Other variables were normally distributed.

Normative data

MEP latency and CMCT values for both muscles are presented in Table 1.

**Table 1:** Normative MEP latency and CMCT values obtained from ADM and TA muscles ADM: Abductor digiti minini; TA: Tibialis anterior; MEP: Motor-evoked potential; CMCT: Central motor conduction time

	Rest		Voluntary Contraction		MI	
	MEP Latency (ms)	CMCT (ms)	MEP Latency (ms)	CMCT (ms)	MEP Latency (ms)	CMCT (ms)
ADM	23.3 ± 1.4	9.2 ± 1.3	20.6 ± 1.3	6.4 ± 1.1	22.4 ± 1.4	8.3 ± 1.2
TA	30.7 ± 2.8	12.9 ± 2.7	28.1 ± 2.1	10.2 ± 1.8	29.7 ± 2.3	12.0 ± 2.3

ANOVA

For both muscles, session (rest x voluntary conduction x MI) was a significant factor for MEP latency (F(2,40) = 21.35, p < 0.0001, $\eta^{2}_{p}$ = 0.18, F(2,40) = 53.22, p < 0.0001, $\eta^{2}_{p}$ = 0.44 for TA and ADM muscles, respectively), CMCT (F(2,40) = 27.22, p < 0.0001, $\eta^{2}_{p}$ = 0.21, F(2,40) = 49.99, p < 0.0001, $\eta^{2}_{p}$ = 0.51 for TA and ADM muscles, respectively) and MEP amplitude (F(2,36) = 79.28, p < 0.0001, $\eta^{2}_{p}$ = 0.68, F(2,40) = 66.75, p < 0.0001, $\eta^{2}_{p}$ = 0.55 for TA and ADM muscles, respectively).

Cortical Motor-Evoked Potential Latency

**Figure 1:**
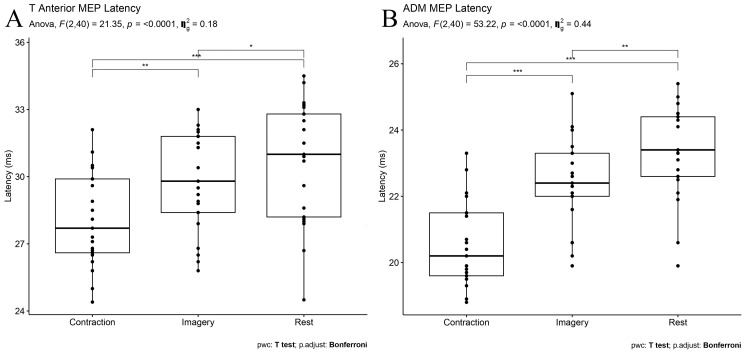
MEP latencies of (A) TA and (B) ADM muscles during rest, MI, and voluntary contraction A repeated ANOVA test revealed that the session (rest x voluntary conduction x MI) was a significant factor in MEP latency for both muscles. P value < 0.05 is considered significant. *p = 0.01, **p = 0.001, ***p < 0.001 MEP: Motor-evoked potential; TA: Tibialis anterior; ADM: Abductor digiti minimi; MI: Motor imagery

For TA and ADM muscles, cortical MEP latency obtained during MI (p = 0.01, p = 0.01, respectively) and mild contraction (p < 0.001, p < 0.001, respectively) was significantly shorter than at rest, respectively. In addition, cortical MEP latency during contraction was significantly shorter compared to the imagery state for TA and ADM muscles (p = 0.001, p < 0.001, respectively) (Figure 1).

Central Motor Conduction Time

**Figure 2:**
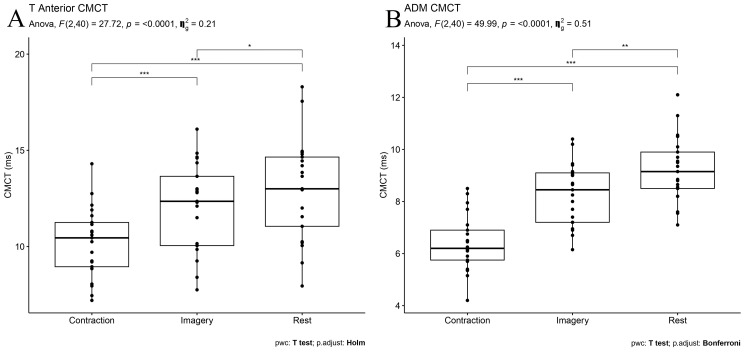
CMCT values of (A) TA and (B) ADM muscles during rest, MI, and voluntary contraction A repeated ANOVA test revealed that the session (rest x voluntary conduction x MI) was a significant factor in CMCT for both muscles. P value < 0.05 is considered significant. *p = 0.01, **p = 0.001, ***p < 0.001 CMCT: Central motor conduction time; TA: Tibialis anterior; ADM: Abductor digiti minimi; MI: Motor imagery

CMCT obtained at rest in both muscles was significantly prolonged compared to the MI (p = 0.01 and p = 0.001 for TA and ADM muscles, respectively) and contraction states (p < 0.001 and p < 0.001 for TA and ADM muscles, respectively). In addition, CMCT obtained in the MI state was significantly prolonged in both muscles compared to the voluntary contraction state (p < 0.001 and p < 0.001 for TA and ADM muscles, respectively) (Figure 2).

Motor-Evoked Potential Amplitude

**Figure 3:**
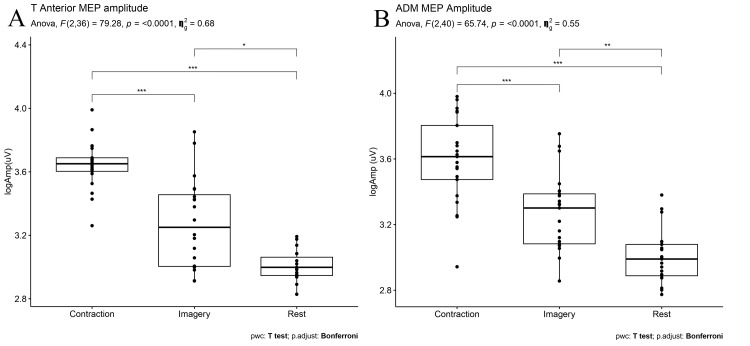
Logarithmic MEP amplitudes of (A) TA and (B) ADM muscles muscles during rest, MI, and voluntary contraction A repeated ANOVA test revealed that the session (rest x voluntary conduction x MI) was a significant factor in MEP amplitude for both muscles. P value < 0.05 is considered significant. *p = 0.01, **p = 0.001, ***p < 0.001 MEP: Motor-evoked potential; TA: Tibialis anterior; ADM: Abductor digiti minimi; MI: Motor imagery

MEP amplitudes were significantly higher in both muscles during MI (p = 0.01 and p = 0.001 for TA and ADM muscles, respectively) and voluntary contraction (p < 0.001 and p < 0.001 for both TA and ADM muscles, respectively) compared to rest. Additionally, MEP amplitudes obtained during voluntary contraction in both muscles were significantly higher than in the MI condition (p < 0.001 and p < 0.001 for both TA and ADM muscles, respectively) (Figure 3).

Correlation analysis

Tibialis Anterior​​​​​​​ Muscle

**Figure 4:**
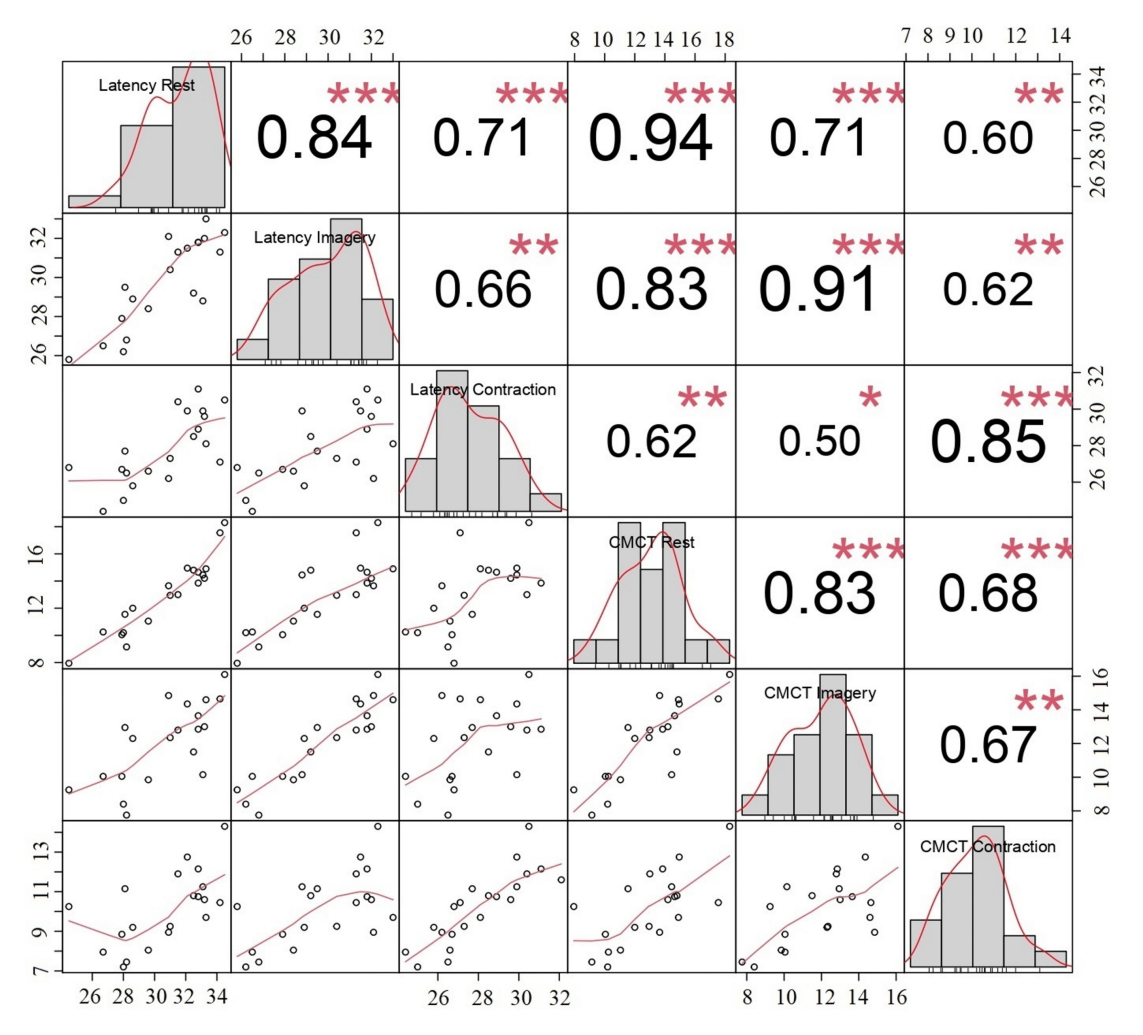
Correlation of MEP latencies and CMCT values during rest, MI, and voluntary contraction in TA muscle Pearson's correlation analysis detected correlations between MEP latencies and CMCT in rest, MI, and contraction states in the TA muscle. P value < 0.05 is considered significant. *p < 0.05, **p < 0.01, ***p < 0.001 MEP: Motor-evoked potential; CMCT: Central motor conduction time; MI: Motor imagery; TA: Tibialis anterior

There was a significant correlation between cortical MEP latency and all CMCT parameters obtained in the three different protocols. The correlation was generally strong or very strong (Figure 4).

Abductor Digiti Minimi ​​​​​​​Muscle

**Figure 5:**
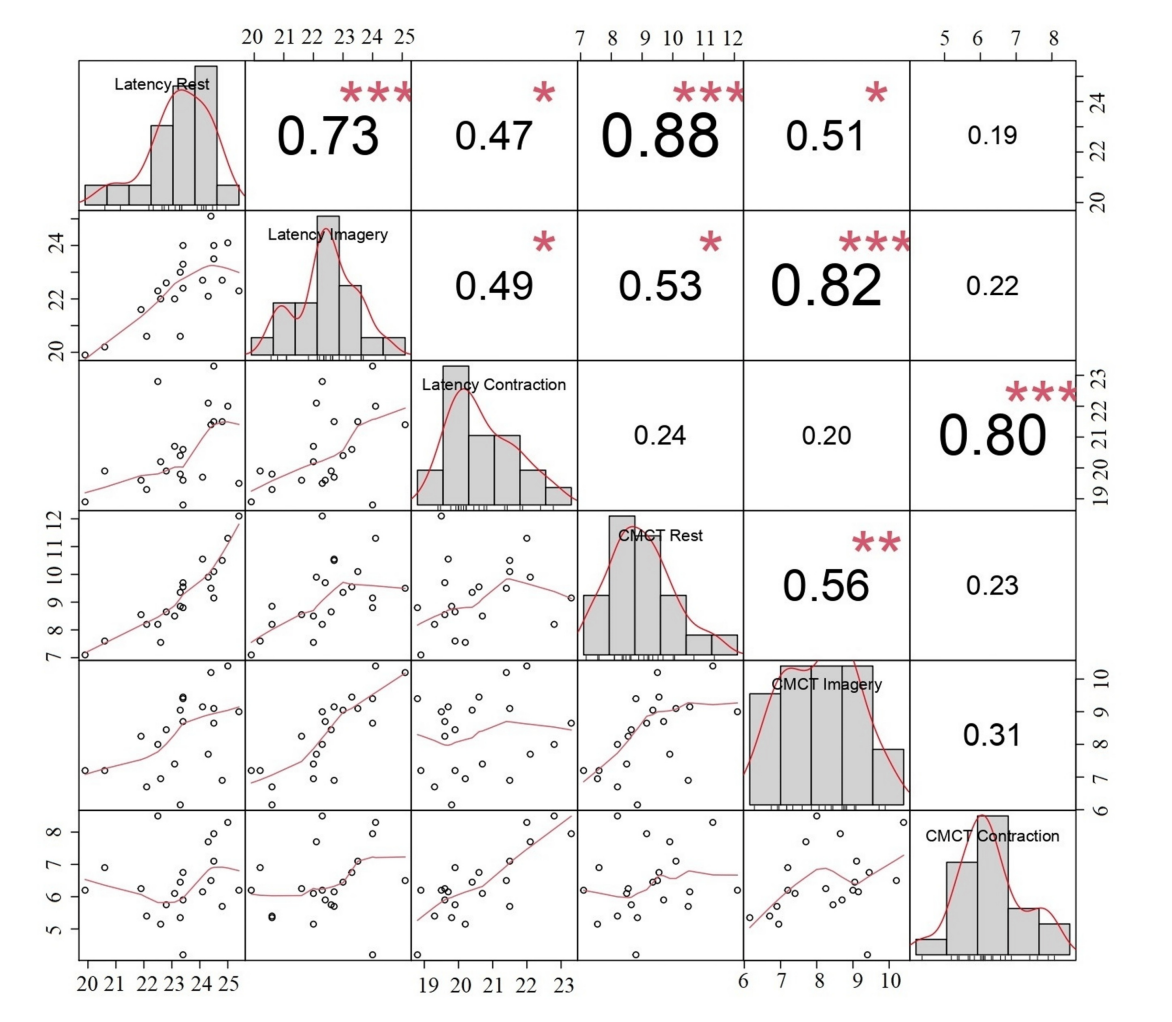
Correlation of MEP latencies and CMCT values during rest, MI, and voluntary contraction in ADM muscle Pearson's correlation analysis detected correlations between MEP latencies and CMCT in rest, MI, and contraction states in the ADM muscle. P value < 0.05 is considered significant. *p < 0.05, **p < 0.01, ***p < 0.001 MEP: Motor-evoked potential; CMCT: Central motor conduction time; MI: Motor imagery; ADM: Abductor digiti minimi

The striking finding in the ADM muscle was that cortical MEP latency and CMCT obtained with contraction were not correlated with cortical MEP latency and CMCT values obtained with MI and at rest (Figure 5). In addition, the correlation strength between the variables was generally weaker than that obtained in the TA muscle.

## Discussion

The remarkable finding of this study is that MI was shown for the first time as a physiological factor affecting CMCT such as voluntary contraction. Previous functional magnetic resonance imaging (fMRI) and TMS studies have shown the ability of imagery to alter cortical adaptability. MI primarily recruits a network of premotor-parietal cortical regions, the thalamus, putamen, and cerebellum whereas movement execution engages sensorimotor-premotor areas, the thalamus, putamen, and cerebellum [9,10]. The result of an fMRI study with hand, foot muscles, and tongue showed that the content of the mental motor image, the body part, is reflected in the pattern of motor cortical activation [11]. Since both MI and movement execution share common neural structures, MI has been used for skill acquisition, sports performance enhancement, and rehabilitation (for a recent review) [9,12,13,14].

The results of a study investigating the relationship between MI and lateralisation indicate that the MI task of the extremity on the opposite side of the hemisphere stimulated with TMS increases excitability. The MI of the same side extremity does not change the corticospinal excitability [15]. In another study, MI of right-hand finger movement in right-handed subjects caused more increase in corticospinal activity and MEP amplitude than MI of the same movement in the left hand. No significant difference was found in MEP amplitude increase caused by MI of right or left-hand finger movement in left-handed subjects [16].

The result of a near-infrared spectroscopy (NIRS) study comparing the effect of MI and motor execution on motor cortex (M1) activity showed an increase in M1 activity in both MI and motor execution compared to the resting state. However, MI-related activation was slower and smaller than motor execution [17]. Another fNIRS study showed that the activation obtained with MI was slower than motor execution, and there were significant differences in the spatial distribution of activation between execution and MI, in agreement with the study of Wriessnegger et al. [18]. We think that the findings of this study, in accordance with the findings of the above two studies, are important in demonstrating electrophysiologically that increased corticospinal activity compared to rest is more prominent in the execution of the movement than in the imagination of that movement.

Another striking finding of this study was the correlation between the electrophysiological findings obtained during the execution of the movement related to MI. The results of correlation analyses revealed that the correlation between the variables of MEP latency and CMCT measurements obtained during the execution of the movement related to MI differed according to the muscle. In the TA muscle, CMCT and MEP latencies obtained in voluntary contraction were strongly correlated with CMCT and MEP latencies obtained during MI. Such a correlation was absent in the ADM muscle. This finding may be related to the characteristics of the motor output, i.e., movement, of the two muscles. In creatures at the top of the evolutionary ladder, including humans, there are direct connections from the cortex to the alpha motor neuron [19]. Thus, a large part of the corticospinal tract projects to the motor regions of the spinal cord and brainstem to regulate fine motor movements in humans [20]. ADM is the distal hand muscle responsible for fine and complex movements [21]. Since it is responsible for more complex and organised movements than the TA muscle, the imagery of a complex motor movement will also be complex, and the correspondence between MI and motor output in this muscle may not be as consistent as in the TA muscle. Examining the relationship between MI and motor movement in diseases, such as stroke and motor neuron disease with corticospinal involvement, may provide information that will allow us to test this hypothesis.

This study has some limitations. Since MI was not quantified in this study, the correlation between imagery power and electrophysiological parameters was not examined. Since spinal excitability measurement (such as H reflex or F response) was not performed, the relationship between the corticospinal excitability change detected during MI and mild muscle and spinal excitability could not be investigated.

## Conclusions

The study demonstrated that the execution of the movement associated with MI exhibited distinct characteristics according to the muscle group involved. Results showed that MI evokes a contraction-like effect on CMCT and MEP latency. The findings of this study indicate that MI should be considered a physiological phenomenon that may influence CMCT. One may posit that MI represents a state situated between the resting and motor activity states with regard to corticospinal activity.
